# AMP-Conjugated Quantum Dots: Low Immunotoxicity Both In Vitro and In Vivo

**DOI:** 10.1186/s11671-015-1100-3

**Published:** 2015-11-05

**Authors:** Tongcheng Dai, Na Li, Lu Liu, Qin Liu, Yuanxing Zhang

**Affiliations:** State Key Laboratory of Bioreactor Engineering, East China University of Science and Technology, 130 Meilong Road, Shanghai, 200237 China; Shanghai Collaborative Innovation Center for Biomanufacturing Technology, Shanghai, 200237 China; Department of Chemistry, College of Science, University of Shanghai for Science and Technology, Shanghai, 200093 China

**Keywords:** AMP, Quantum dots, Immunotoxicity, In vivo

## Abstract

Quantum dots (QDs) are engineered nanoparticles that possess special optical and electronic properties and have shown great promise for future biomedical applications. In this work, adenosine 5′-monophosphate (AMP), a small biocompatible molecular, was conjugated to organic QDs to produce hydrophilic AMP-QDs. Using macrophage J774A.1 as the cell model, AMP-QDs exhibited both prior imaging property and low toxicity, and more importantly, triggered limited innate immune responses in macrophage, indicating low immunotoxicity in vitro. Using BALB/c mice as the animal model, AMP-QDs were found to be detained in immune organs but did not evoke robust inflammation responses or obvious histopathological abnormalities, which reveals low immunotoxicity in vivo. This work suggests that AMP is an excellent surface ligand with low immunotoxicity, and potentially used in surface modification for more extensive nanoparticles.

**Electronic supplementary material:**

The online version of this article (doi:10.1186/s11671-015-1100-3) contains supplementary material, which is available to authorized users.

## Background

Quantum dots (QDs) have garnered a great deal of attention because of their attractive photophysical properties, including a high photoluminescence quantum yield (PLQY), superior photostability and a narrow and symmetric emission spectrum coupled with a broad and continuous excitation spectrum [1–4]. CdSe nanocrystals are among the brightest, best-studied and most widely available quantum dots used for bioimaging [5], and they have been successfully prepared via the organometallic route with a PLQY as high as 85 % [6]. However, organic synthesised QDs are typically hydrophobic in nature and thus cannot be used directly in biological applications. Post-treatment is therefore required to render QDs with aqueous dispersibility [7, 8]. The most common strategy for doing so is to engineer QDs via surface modifications [9–11]. However, surface coatings may lead to a significant size increase beyond the desired range [12, 13] and a decrease of the PLQY [14]. More distinctly, different surface ligands have reportedly caused additional toxicity by interacting with host’s innate immune system and evoking significant inflammatory effects [15–17]. For example, the proinflammatory effects of poly(ethyleneglycol) (PEG)ylated CdSe/ZnS QDs were reported as being strongly associated with the functional groups (-COOH, -NH_2_, -OH and -OCH_3_) at the end of the PEG chain [18]. It is no doubt that excessive immune responses will, to a certain degree, hamper QDs’ use in biological living systems.

Recently, Liu et al. reported the synthesis of adenosine 5′-monophosphate (AMP) modified CdSe/CdS/ZnS QDs (AMP-QDs) [19], which maintained a high PLQY (one nearly identical to that of the original oil-soluble QDs) and a small hydrodynamic size (~7.1 nm), and also showed excellent stability even under various extreme conditions (pH ranging from 3 to 13 and NaCl concentrations up to 5 M). All of these features make AMP-QDs potentially great tools for biological imaging. Because AMP is an universal molecule in biological systems, we hypothesised that AMP coatings could, to a certain degree, assist QDs in evading host’s innate immune system and thus render QDs with a low immunotoxicity.

In this work, AMP-modified quantum dots (AMP-QDs) were prepared as described by Liu et al. [19]. To further explore the potential use of AMP-QDs in biological systems, we investigated their imaging behaviour in macrophage and evaluated their immunotoxicity both in vitro and in vivo.

Using J774A.1 as the macrophage cell model, we first investigated AMP-QDs’ imaging property by confocal laser scanning microscopy (CLSM) and cytotoxicity by 3-(4,5-dimethylthiazol-2-yl)-2,5-diphenyltetrazolium bromide (MTT). Subsequently, acute inflammation responses in macrophage to AMP-QDs were assayed by real-time PCR (RT-PCR). Furthermore, using BALB/c mice as the animal model, blood circulation and biodistribution of AMP-QDs were studied by measuring Cd content, which was quantified with inductively coupled plasma-mass spectrometry (ICP-MS). Proinflammatory responses in immune organs to AMP-QDs were conducted by measuring key cytokines transcription levels including tumour necrosis factor-α (TNF-α) and interleukin (IL)-1β. Histopathological assay of immune organs was evaluated by haematoxylin and eosin (H&E) staining.

## Methods

### Chemicals

The water-soluble AMP or 3-mercaptopropionic acid (MPA) capped CdSe/CdS/ZnS QDs (AMP/MPA-QDs) were synthesized according to the procedure developed by Liu et al. [19, 20]. Oil phase CdSe/CdS/ZnS QDs with a fluorescence emission of 610 nm were used as the starting materials. All chemicals were obtained from Sigma (St. Louis, MO, USA) unless otherwise noted.

### Characterisation of Quantum Dots

Absorption was measured on a Shimadzu UV-2450 spectrophotometer. Fluorescence emission spectra were obtained by a Cary Eclipse (Varian) fluorescence spectrophotometer. The morphology and size of quantum dots were analysed by transmission electron microscopy (TEM) obtained on a JEOL JEM-1400. The hydrodynamic size of quantum dots was investigated by dynamic light scattering (DLS) with Zetasizer NanoZS Instrument (Malvern Instrument Corporation).

### Cell Lines and Cell Culture

The macrophage cell line J774A.1 was purchased from the China Center for Type Culture Collection (Wuhan, China). J774A.1 cells were cultured in DMEM media supplemented with 10 % foetal bovine serum and were cultured in a 5 % carbon dioxide atmosphere at 37 °C.

### Imaging of QDs in J774A.1 Macrophage Cells

J774A.1 cells were seeded onto sterilised 17-mm-diameter glass coverslips in 12-well plates (1 × 10^5^ cells per well) and incubated for 24 h at 37 °C. Cells were then washed with phosphate buffer saline (PBS) and incubated in a media in the presence of 50 nM AMP/MPA-QDs. After 12 h, they were then washed with PBS and prepared for staining using a fixative solution for 10 min at room temperature, and the nuclei were stained with 4', 6-diamidino-2-phenylindole (DAPI). The slides were imaged with a laser scanning confocal microscope.

### Cell Uptake Efficiency of QDs Measured by ICP-MS

J774A.1 cells were seeded in 12-well plates (1 × 10^5^ cells per well) and incubated for 24 h at 37 °C. Cells were then washed with PBS and incubated in a media in the presence of 50 nM AMP/MPA-QDs. After 12 h, they were then washed with PBS, and the cells were lysed in a 1-ml digest solution (HNO_3_: HCl ratio of 10:1). The intracellular Cd^2+^ content was quantified using inductively coupled plasma-mass spectrometry (ICP-MS) and compared with standards.

### MTT Analysis

Twenty-four hours after cell seeding, J774A.1 cells were incubated with a range of concentrations of AMP/MPA-QDs for 24 or 48 h at 37 °C, and then 10 μl of MTT (5 mg/ml) was added to each well and allowed to incubate for 4 h. Next, 100 μl of 10 % sodium dodecyl sulfate (SDS) solution was added to dissolve the formazan crystals during an additional 4-h incubation. The absorbance of the MTT formazan was determined at A_570 nm_ with a spectrophotometer (SpectraMax M5, Molecular Devices, USA) following noncellular background (i.e., a blank consisting of the complete media, yellow MTT and SDS solution) subtraction. Results are expressed as the percent of MTT conversion activity for the media-treated control cells and are composed of six biological replicates.

### Quantitative RT-PCR Analysis of Gene in Macrophage

To verify the differential receptors and cytokines gene expression in J774A.1 macrophage induced by quantum dots, RT-PCR was performed using the ABI Prism 7500 Detection System (Applied Biosystems, Foster City, CA, USA) with the fluorescent detection dye SYBR Green (Roche, USA) according to the manufacturer’s protocol. The total messenger RNA (mRNA) from cellular samples was extracted using the TRIzol lysis reagent (Invitrogen, USA) according to the manufacturer’s protocol. First-strand cDNA was synthesised from 1 μg of the total extracted mRNA and was utilized as the template for qPCR with gene-specific primers (described in Table 1). Primers were designed using the Primer Premier 5 software. The RT-PCR thermal cycling conditions for all reactions were 95 °C for 15 min followed by 40 cycles of 95 °C for 5 s, 60 °C for 20 s and 72 °C for 20 s. All RT-PCR reactions were performed using three biological replicates, and the data for each sample were expressed relative to the expression levels of β-actin by using the 2^−ΔΔCT^ method [21].

**Table 1:** Primer sequences for the analysed genes in J774A.1 cells

Gene	Forward (5′-3′)	Reverse (5′-3′)
β-actin	TTCTTTGCAGCTCCTTCG	TCGTCGCCCGCGAAGCC
TLR-2	GTCGTTCAAGGAGGTGCG	TGAGGTTTCGGTAAGTTGT
TLR-3	GGAGCCAGAACTGTGCCAAATACTC	TCGCAACGCAAGGATTTTATTTTTT
TLR-4	TCTGGTGGCTGTGGAGAC	TTGTGAGCCACATTGAGT
TLR-5	TGGGGACCCAGTATGCTAACT	CCACAGGAAAACAGCCGAAGT
TLR-7	CTTACCCTTACCATCAAC	TTGGACCCCAGTAGAAC
TLR-9	CGGGAACTGCTACTACAAGA	GGTTGTTATACTTCAGAGAC
MyD88	ACTCGCAGTTTGTTGGATG	TGTAAAGGCTTCTCGGACT
NF-κB	TTCCAGGTGACAGTGCGG	GAGTTCCGGTTTACTCGG
I-κB	GCCCTTCCTCCCTAACTG	GGGTAGCGAACTTGAAAAC
TNF-α	TCTCATTCCTGCTTGTGG	GAGGGTCTGGGCCATAGA
IL-1β	TCGTGCTGTCGGACCCAT	TCCTTTGAGGCCCAAGGCCA
TGF-β	CTC TCCACCTGCAAGACCAT	CTGCCGTACAATTCCAGTGA
MCP-1	TGAGGTGGTTGTGGAAAAGG	CCTGCTGTTCACAGTTGCC

### Animal Experiments

Six-week-old female BALB/c mice were purchased from Shanghai SLAC Laboratory Animal Co., Ltd. The study was approved by the Experimental Animal Management and Ethics Committees of Shanghai Jiaotong University School of Pharmacy. For the AMP-QDs in vivo toxicity experiments, there were six mice per group at each time point for statistical analysis. The mice were intravenously injected with 100 μL of PBS solution as the control group and 100 μL of AMP-QDs solution containing 0.4 nmol QDs as the experimental group. We collected the mouse body weights at indicated time for 60 days. The female BALB/c mice were sacrificed and blood and organs were collected at several time points post-injection.

### Blood Circulation and Biodistribution Experiments

After injection of the AMP-QDs and PBS solution, the mice were sacrificed by exsanguination at various time points. The immune organs and tissues, which included the liver, spleen, kidney and blood, were weighed and then dissolved in 5 ml of a digest solution (HNO_3_:HCl ratio of 10:1) overnight. A microwave digestion system was used to ensure continuous digestion. The mixed solution became clear following digestion, and it was then cooled down at room temperature. Each of the samples was diluted to 10 ml by Milli-Q water. ICP-MS was used to analyse the concentration of Cd^2+^ in each sample.

### Histology Experiments

BALB/c mice were sacrificed. The major immune organs, including the liver, spleen and kidney, were collected and fixed with 10 % buffered formalin following a rinse with PBS. They were then embedded in paraffin, sectioned and finally stained with H&E staining to prepare them for examination by digital microscopy.

### Transcription Analysis of Proinflammation Responses to AMP-QDs in Mice

Total RNAs from the liver, spleen and kidney were extracted by the TRIzol lysis reagent according to the manufacturer’s protocol. The expression of key inflammatory cytokines such as TNF-α and IL-1β was measured using RT-PCR as described in the above method.

### Statistical Analysis

The results were showed in mean ± standard deviation (s.d.). Statistical analysis was measured by two-tailed Student’s *t* test. A difference of *P* < 0.05 was considered to be statistically significant.

## Results and Discussion

### Characterization of Quantum Dots

**Fig. 1:**
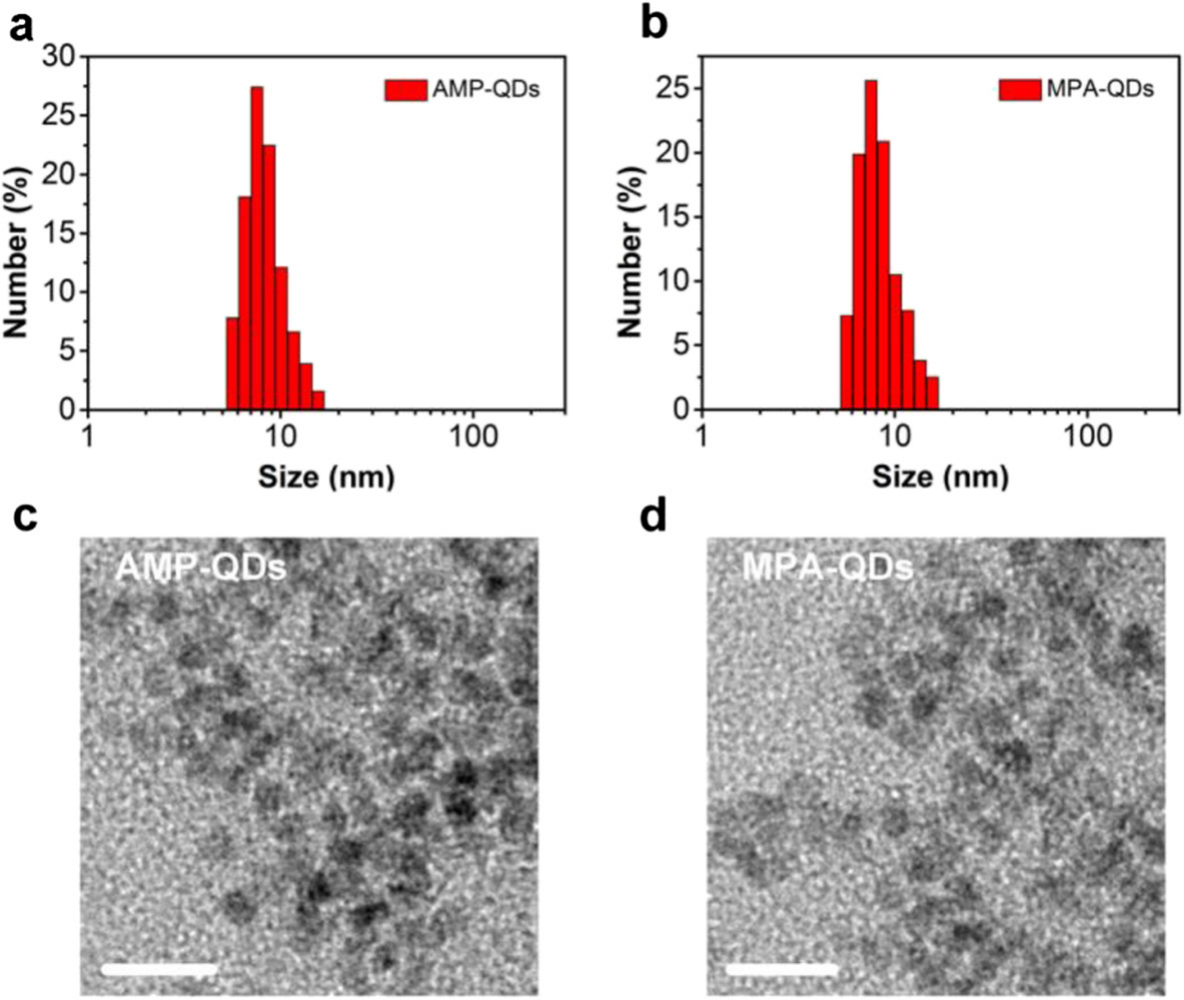
The size and morphology of quantum dots. The hydrodynamic diameter of AMP-QDs (**a**) and MPA-QDs (**b**) were measured by dynamic light scattering (DLS). The corresponding TEM images of AMP-QDs (**c**) and MPA-QDs (**d**) were investigated., *scale bars*: 20 nm

**Fig. 2:**
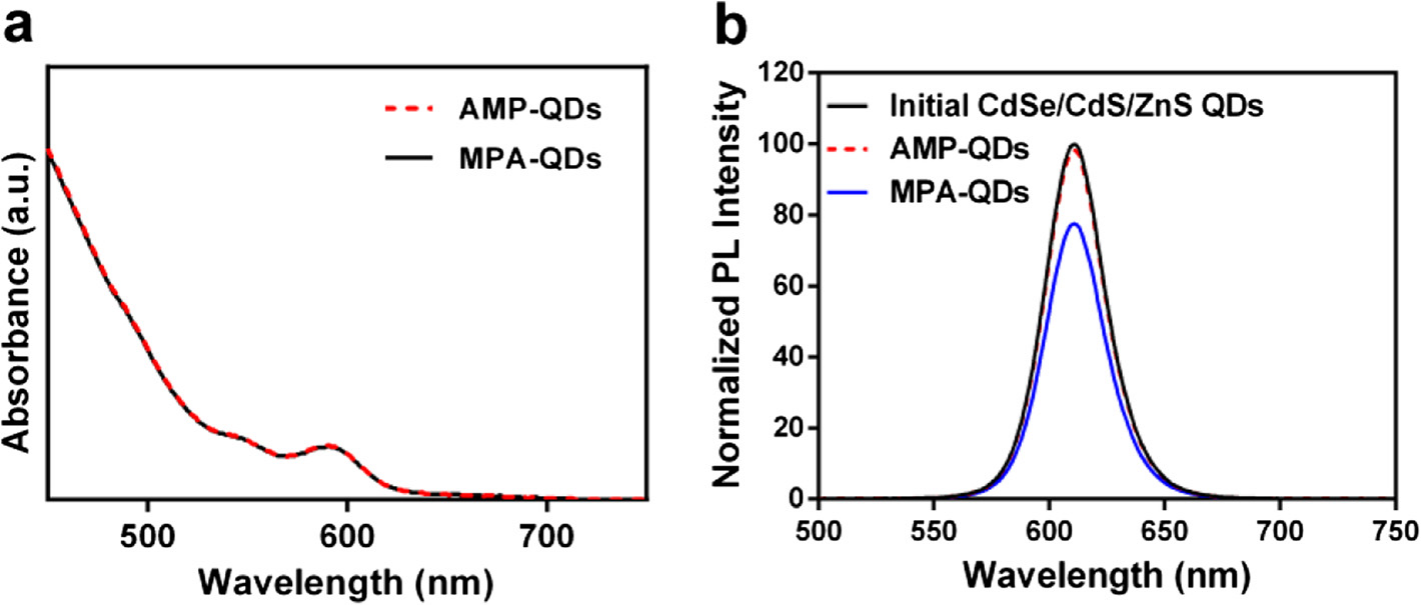
Absorption spectrum (**a**) and fluorescence spectrum (**b**) of quantum dots were tested.

The hydrodynamic diameter of AMP-QDs in water was 8.41 nm (Fig. 1a), similar to the size of MPA-QDs (8.32 nm, Fig. 1b). We further detected the size and distribution of AMP-QDs in more extensive physiological media including 0.01 M PBS and cell culture medium containing 10 % foetal bovine serum, and the results were similar to Fig. 1a (Additional file 1: Figure S2). It indicated that AMP-QDs showed good dispersibility in both PBS and cell culture medium. The TEM images suggest that both AMP-QDs and MPA-QDs were monodispersity (Fig. 1c and d). After phase transfer, AMP-QDs and MPA-QDs showed identical absorption spectral profiles (Fig. 2a). Meanwhile, they also exhibited identical emission spectral profiles to that of original oil quantum dots with an emission peak of 610 nm (Fig. 2b). In the aspect of fluorescent brightness, AMP-QDs preserved high fluorescent brightness (showing almost identical PL efficiencies to that of the original oil quantum dots) (Fig. 2b). However, heavy loss of luminescence brightness of quantum dots was observed in MPA-QDs group under the same condition (Fig. 2b).

### Imaging of Macrophage with AMP-QDs

**Fig. 3:**
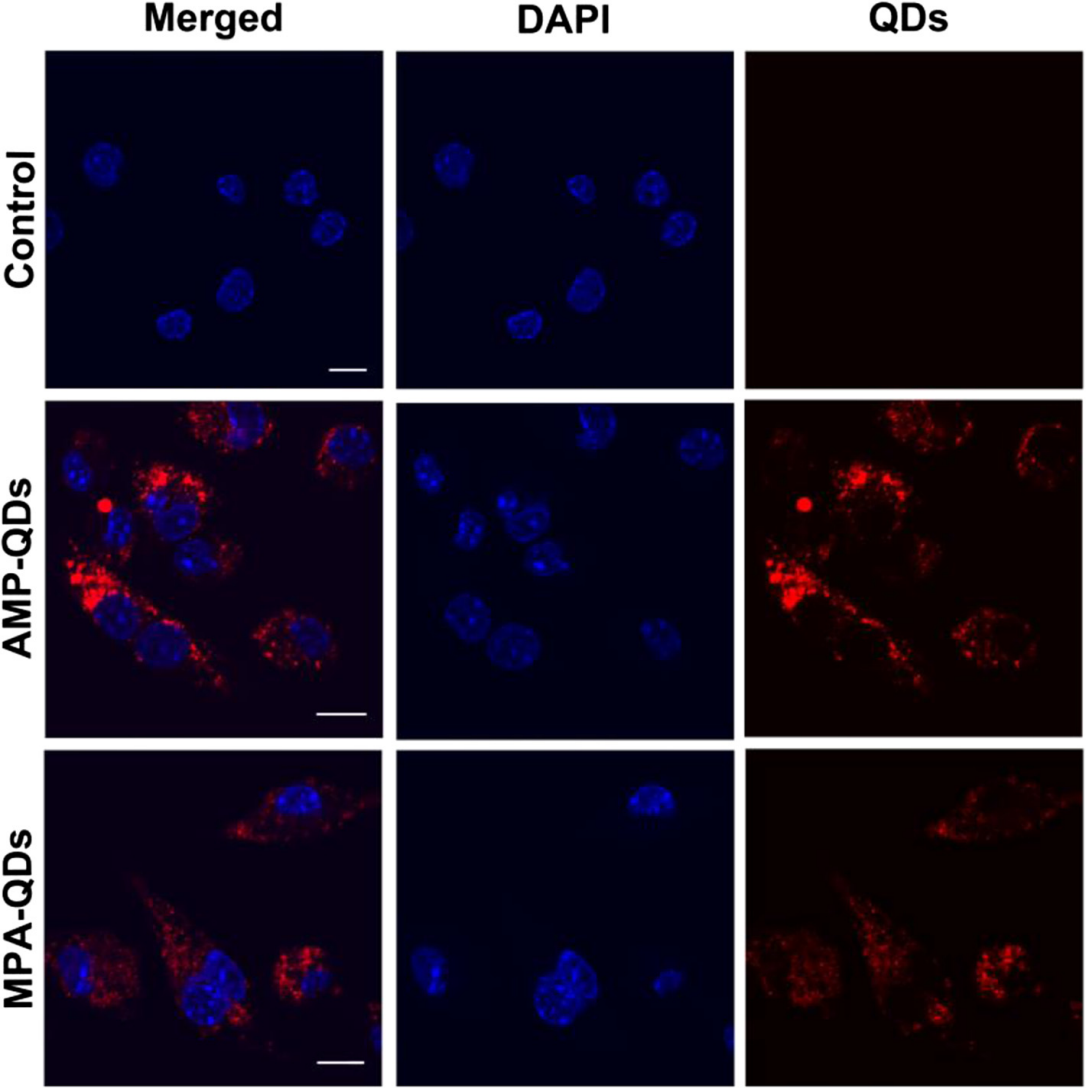
Confocal micrographs of J774A.1 cells incubated with QDs (50 nM) for 12 h were conducted. *Red*: QDs, *Blue*: DAPI. *Scale bars*: 10 μm

Because macrophages are key innate immune effector cells with pivotal roles in the uptake of nanoparticles [22, 23], it is of great interest to investigate the interaction between AMP-conjugated QDs and macrophages. MPA are also small molecule and widely used in QDs surface modification [20]. In this section, MPA-QDs were used as the control model. Upon incubation with murine macrophage-like cell line J774A.1, AMP-QDs were efficiently incorporated into the macrophages and dispersed in the perinuclear and cytoplasmic regions (Fig. 3). We further quantified the AMP-QDs and MPA-QDs inside the macrophage by intracellular Cd^2+^ detection experiment, and the data confirmed that AMP-QDs and MPA-QDs have similar internalisation efficiency in macrophage (Additional file 1: Figure S1). In addition, AMP-QDs possessed a high PLQY, so they exhibited a more intense fluorescence in the cells than the widely used MPA-QDs under the same conditions (Fig. 3). These data indicated that AMP, as a surface ligand for QDs, is superior to MPA in cell imaging.

### Cytotoxicity Analysis of AMP-QDs by MTT

**Fig. 4:**
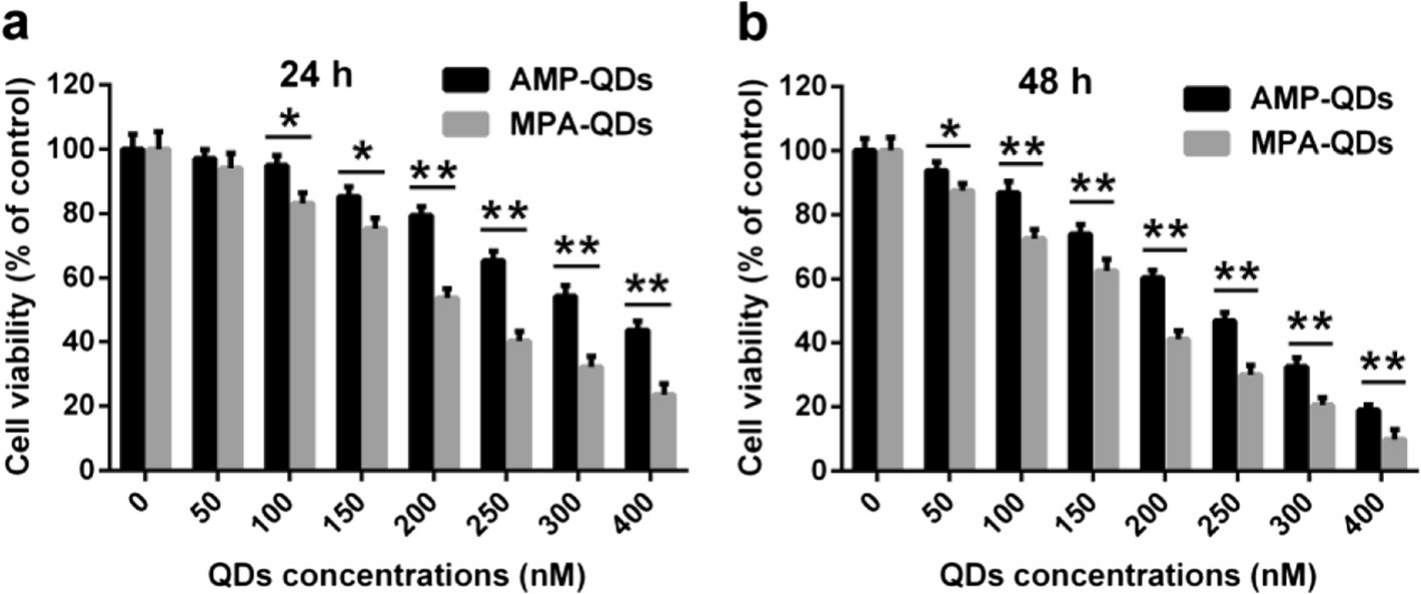
Relative cell viability analysis of J774A.1 cells after incubation of indicated QDs for 24 (**a**) and 48 h (**b**). *Error bars* represent s.d. (*n* = 6). *P < 0.05, **P < 0.01 (two-tailed Student’s *t* test)

To evaluate cell toxicity in J774A.1 cells after cellular uptake of QDs, the cell viability was investigated by MTT assay at 24 or 48 h post-incubation of AMP/MPA-QDs with untreated cells as controls. The results are shown in Fig. 4. Both AMP-QDs and MPA-QDs display time- and dose-dependent manner in their cytotoxicity to J774A.1 cells. Results indicated that cell growth was not significantly inhibited by AMP-QDs at 100 nM, and the IC_50_ value for the AMP-QDs was 336 nM at 24 h (Fig. 4a) and 234 nM at 48 h (Fig. 4b), which are obviously higher than that of the corresponding widely used MPA-QDs. These results demonstrated that AMP-QDs were less toxic than MPA-QDs, hinting that different surface modifications of nanoparticles will affect their cytotoxicity.

### Acute Inflammation Responses in Macrophage to AMP-QDs

In previous study, nanoparticles can induce acute inflammation in immune cells [24]. In order to get more conclusive information about the immune response profile elicited by AMP-QDs in macrophages, the transcriptional levels of acute inflammation response genes at 4 h after adding AMP-QDs into J774A.1 cell cultures were determined by RT-PCR method.

**Fig. 5:**
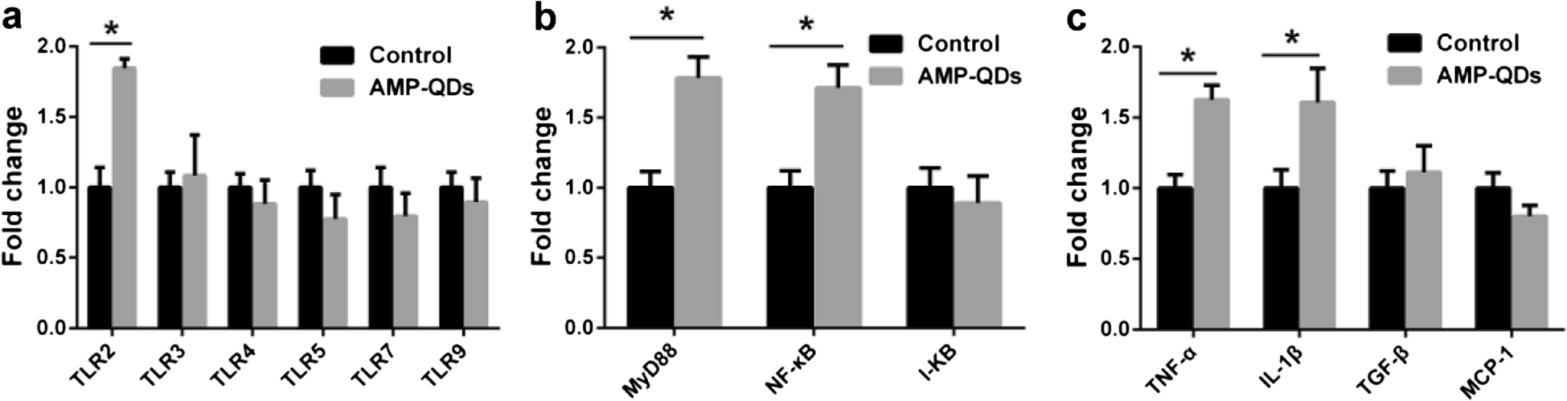
Analysis of TLR signalling pathway-related gene expression in macrophage J774A.1 incubated with AMP-QDs (100 nM) for 4 h by RT-PCR. **a** TLRs gene analysis, **b** NF-κB signalling pathway-related genes detection and **c** cytokines and chemokine measurement. *Error bars* represent s.d. (*n* = 3). *P < 0.05 (two-tailed Student’s *t* test). Exceeded thresholds of two-fold induction or 0.5-fold suppression were considered as significant variation comparing AMP-QDs and control groups [26]

Toll-like receptors (TLRs) are important pattern recognition receptor family for the detection of foreign nanomaterials and subsequent induction of innate immune process [25]. As showed in Fig. 5a, the expression levels of TLR2 were increased by 1.84-fold, while the other TLRs, including TLR3, TLR4, TLR5, TLR7 and TLR9, kept unchanged or minor reduced. This result indicated that TLR2 may be the receptor responsible for recognizing AMP-QDs in macrophage.

Upon activation, TLRs recruit adaptor proteins such as myeloid differentiating factor 88 (MyD88) and trigger downstream signalling proteins such as NF-κB to regulate subsequent inflammation responses. NF-κB is a cytosolic transcription factor binding to nuclear DNA and activating transcription of target genes. In the classical activation pathway, activation of NF-κB is controlled by its inhibitory subunit, inhibitor of NF-κB (I-κB), which prevents NF-κB subunits from leaving the cytosol. As showed in Fig. 5b, slight upregulation of MyD88 (1.78-fold) combined with NF-κB (1.71-fold) and downregulation of I-κB (0.89-fold) were found in AMP-QDs-treated group, compared to the control group. This result suggest that AMP-QDs, followed by activating TLR2, further transduced the signals to MyD88 and NF-κB pathway.

Activated NF-κB pathway could induce proinflammatory cytokines including IL-1β and TNF-α [26], and eventually result in diverse cellular inflammatory responses including secretion of cytokines. Results are showed in Fig. 5c. In the cells treated by AMP-QDs, the mRNA expression of TNF-α and IL-1β are slightly increased by 1.62- and 1.60-fold, and the expression levels of TGF-β and MCP-1 are nearly not changed. These data revealed that AMP-QDs induced a low inflammation level in macrophage, while MPA-QDs could highly improve inflammation levels [27].

Together, we profiled the acute inflammation responses for AMP-QDs in macrophage, which involve the cascade activation from TLR2 to MyD88/NF-κB pathway then to proinflammatory cytokines. Our data proved that AMP-QDs orchestrated a mild inflammatory response in macrophage, which leads to a low level of immunotoxicity.

### Blood Circulation and Biodistribution of AMP-QDs in Mice

**Fig. 6:**
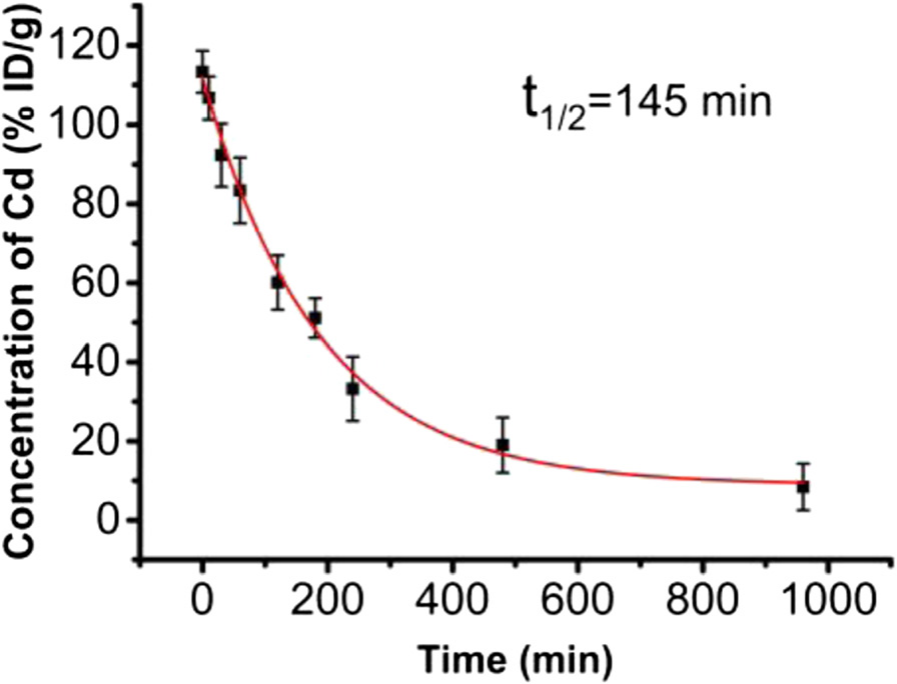
The blood circulation curve of AMP-QDs. The circulation half-life was determined to be 145 min by a method reported previously [27]. *Error bars* represent s.d. (*n* = 6)

To understand the behaviour of AMP-QDs in living mice, we studied their blood clearance and tissue biodistribution following intravenous administration to BALB/c mice with a dosage of 0.4 nmol per mouse. AMP-QDs in the blood were quantified over time by ICP-MS (Fig. 6). The half-life of AMP-QDs in the bloodstream was 145 min, which is significantly shorter than that of the much more widely used poly(ethyleneglycol) (PEG)ylated QDs [28]. It suggested that AMP-QDs exhibited rapid clearance from blood circulation.

**Fig. 7:**
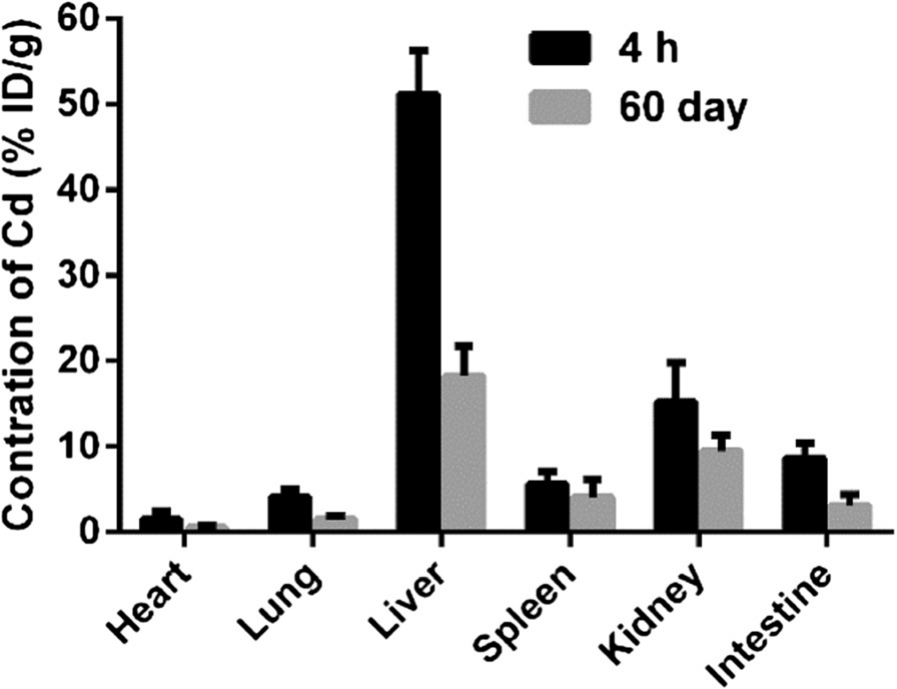
Biodistribution of AMP-QDs treated mice at 4-h and 60 - d analysed by ICP-MS. *Error bars* represent s.d. (*n* = 6)

To further track the trace of AMP-QDs in mice, female BALB/c mice were sacrificed at 4-h and 60-day i.v. of AMP-QDs, and various organs including the heart, lung, liver, spleen, kidney and intestine were collected. The concentrations of AMP-QDs in different organs were measured by ICP-MS. As showed in Fig. 7, at 4-h post-injection, AMP-QDs were found to be intensely detained in immune-related organs, including the liver at 51.12 % ID/g, the kidney at 15.21 % ID/g and the spleen at 5.61 % ID/g. Sixty days after the injection, residual AMP-QDs significantly decreased in the liver by 64.12 %, in the spleen by 26.74 % and in the kidney by 37.19 %. Since AMP-QDs were largely accumulated in the liver, spleen and kidney, it is highly necessary to investigate whether AMP-QDs could cause in vivo immunotoxicity in mice.

### Proinflammatory Responses in Mice to AMP-QDs

**Fig. 8:**
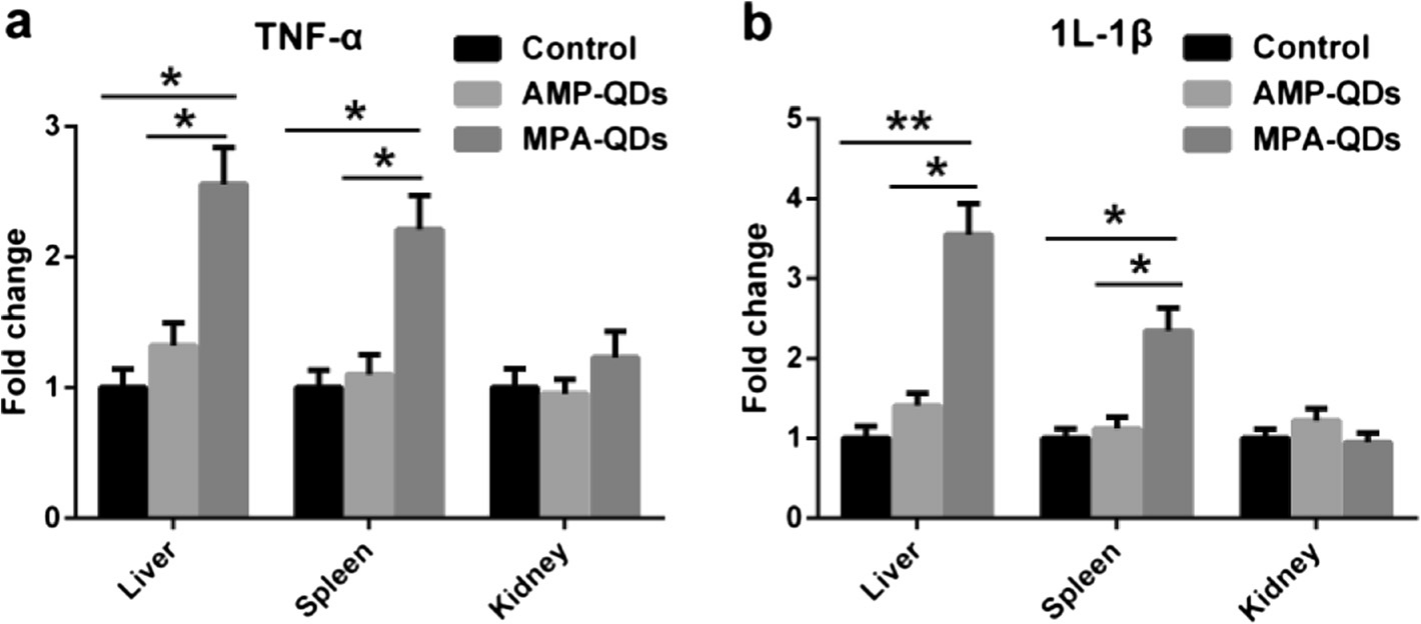
Proinflammatory cytokines TNF-α (**a**) and IL-1β (**b**) levels were measured by RT-PCR in the main immune organs including the liver, spleen and kidney from AMP/MPA-QDs treated and control groups 4 h post-injection. *Error bars* represent s.d. (*n* = 6). *P < 0.05, **P < 0.01 (two-tailed Student’s *t* test). Exceeded thresholds of two-fold induction or 0.5-fold suppression were considered as significant variation comparing experimental groups and control groups [26]

The proinflammatory responses to AMP/MPA-QDs in vivo were quantified by measuring the TNF-α and IL-1β levels in the immune organ samples 4 h post-injection. There were no obvious changes in either the TNF-α or IL-1β levels of AMP-QDs group compared to those of the control group (Fig. 8). However, significant upregulation of TNF-α and IL-1β levels in the liver and spleen was observed in MPA-QDs group compared to AMP-QDs or control group (Fig. 8). It indicated that MPA-QDs induced a more robust proinflammatory activity than AMP-QDs in the liver and spleen.

### Histology Analysis

**Fig. 9:**
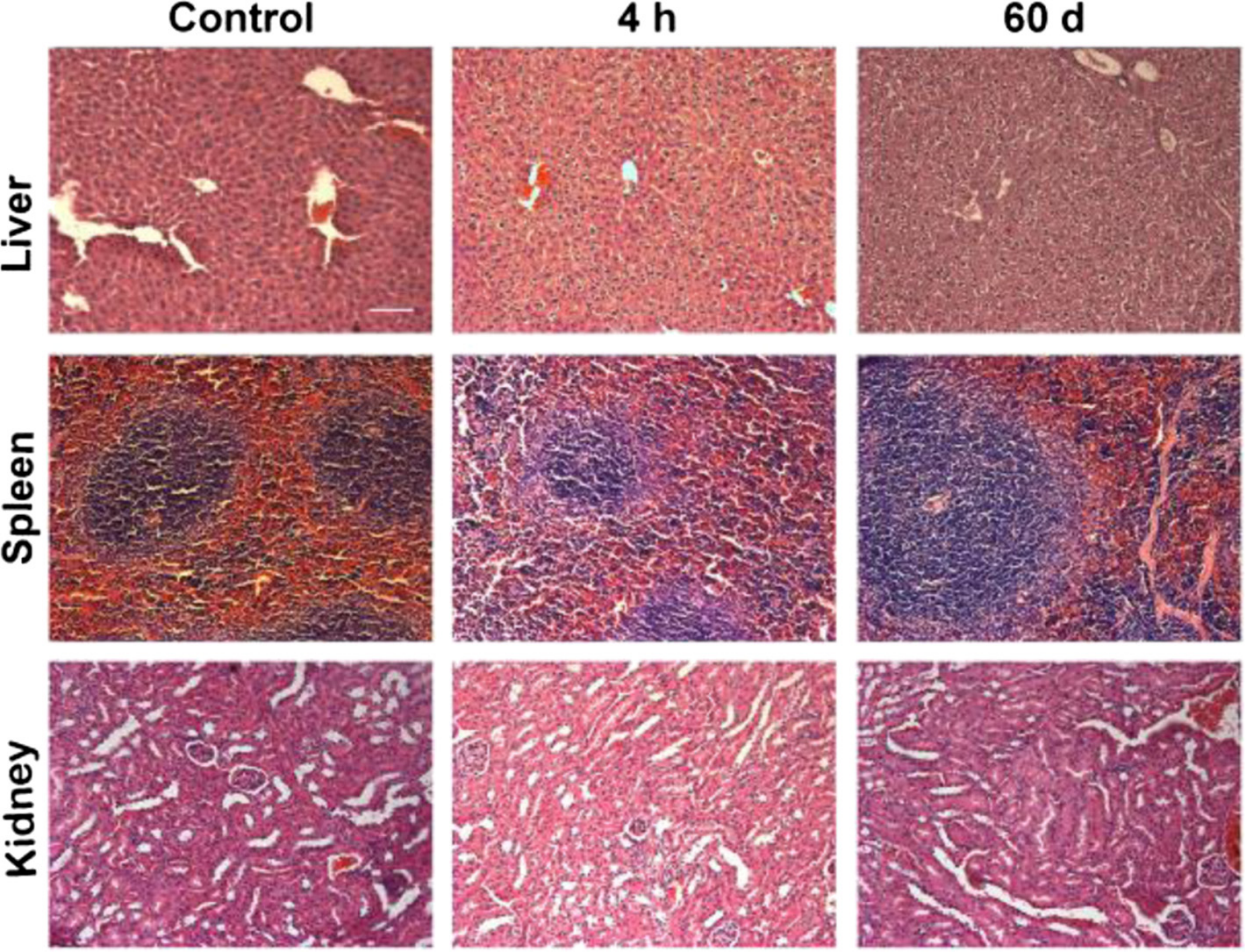
Representative H&E stained images of major immune organs including the liver, spleen and kidney collected from AMP-QDs treated mice and control mice at various time points via intravenous injection. The dose of AMP-QDs was 0.4 nmol. No obvious organ damage or lesion was observed for AMP-QDs-treated mice

Histological analysis of the major immune organs demonstrated that all the tested organs exhibited no apparent histopathological abnormalities or lesions (Fig. 9). Normal hepatocytes in the liver samples were observed, and there were no signs of inflammatory lesions. No hyperplasia was detected in the spleen. The glomerulus structure in the kidney section was clearly observed. These data suggest that AMP-QDs caused no obvious inflammation damage to immune organs.

### Body Weight Measurement

**Fig. 10:**
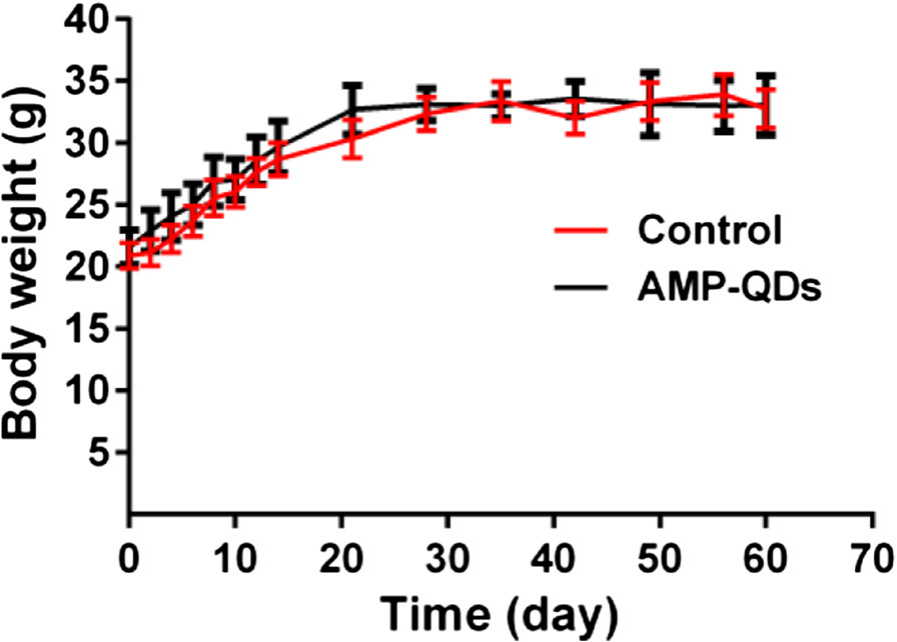
Body weight changes of the mice treated with AMP-QDs. Mice in each group (*n* = 6) were administered with 0.4 nmol AMP-QDs or equal volume of PBS (control) via tail vein for 60 days. The body weights of the mice were measured at indicated time point. *Error bars* represent s.d. (*n* = 6)

Fluctuation in body weight is considered as an effective indicator for qualitatively assessing in vivo toxicity of nanoparticles. The AMP-QDs in PBS were administered to six BALB/c mice through tail vein injection, while another six mice with injection of PBS were set as controls. The effect of AMP-QDs on the body weight of the mice was evaluated. As shown in Fig. 10, the body weights of the control set and the AMP-QDs set of BALB/c mice maintained similar increasing trends over 60 days. It revealed that AMP-QDs did not induce perceivable interference on the growth of mice.

## Conclusions

In this study, AMP, a small molecule universal to biological systems, was conjugated to oil QDs to synthesize hydrophilic AMP-QDs. AMP-conjugated QDs were shown to have prior imaging property, and more essentially a low immunotoxicity both in vitro and in vivo. Our results suggested that AMP-based surface conjugation might be applied as a general strategy to endow nanoparticles with more desirable biocompatibility.

## Additional file

Additional file 1: Figure S1. Intracellular Cd^2+^ concentrations in macrophage J774A.1 cells incubated with quantum dots (50 nM) for 12 h were measured. Data are presented as means ± s.d. (*n* = 6). **Figure S2.** Colloidal stability of AMP-QDs in physiological media. AMP-QDs were incubated for 24 h in 0.01 M PBS (a) or cell culture medium containing 10 % foetal bovine serum, (b) and their size distributions were measured by a dynamic light scattering (DLS) with Zetasizer NanoZS Instrument. The hydrodynamic size of AMP-QDs in 0.01 M PBS (a) and cell culture medium (b) were determined to be 8.49 nm and 8.56 nm, respectively.

## References

[1] Alivisatos P (2004). The use of nanocrystals in biological detection. Nat Biotechnol.

[2] Dubertret B, Skourides P, Norris DJ, Noireaux V, Brivanlou AH, Libchaber A (2002). In vivo imaging of quantum dots encapsulated in phospholipid micelles. Science.

[3] Michalet X, Pinaud FF, Bentolila LA, Tsay JM, Doose S, Li JJ (2005). Quantum dots for live cells, in vivo imaging, and diagnostics. Science.

[4] Valizadeh A, Mikaeili H, Samiei M, Farkhani SM, Zarghami N, Kouhi M (2012). Quantum dots: synthesis, bioapplications, and toxicity. Nanoscale Res Lett.

[5] Ye L, Yong KT, Liu L, Roy I, Hu R, Zhu J (2012). A pilot study in non-human primates shows no adverse response to intravenous injection of quantum dots. Nat Nanotechnol.

[6] Qu L, Peng X (2002). Control of photoluminescence properties of CdSe nanocrystals in growth. J Am Chem Soc.

[7] Gerion D, Pinaud F, Williams SC, Parak WJ, Zanchet D, Weiss S (2001). Synthesis and properties of biocompatible water-soluble silica-coated CdSe/ZnS semiconductor quantum dots. J Phys Chem B.

[8] Pellegrino T, Manna L, Kudera S, Liedl T, Koktysh D, Rogach AL (2004). Hydrophobic nanocrystals coated with an amphiphilic polymer shell: a general route to water soluble nanocrystals. Nano Lett.

[9] Duncan R (2006). Polymer conjugates as anticancer nanomedicines. Nat Rev Cancer.

[10] Qian X, Peng XH, Ansari DO, Yin-Goen Q, Chen GZ, Shin DM (2008). In vivo tumor targeting and spectroscopic detection with surface-enhanced Raman nanoparticle tags. Nat Biotechnol.

[11] Li C, Ji Y, Wang C, Liang S, Pan F, Zhang C (2014). BRCAA1 antibody- and Her2 antibody-conjugated amphiphilic polymer engineered CdSe/ZnS quantum dots for targeted imaging of gastric cancer. Nanoscale Res Lett.

[12] Ballou B, Ernst LA, Andreko S, Harper T, Fitzpatrick JA, Waggoner AS (2007). Sentinel lymph node imaging using quantum dots in mouse tumor models. Bioconjug Chem.

[13] Peer D, Karp JM, Hong S, Farokhzad OC, Margalit R, Langer R (2007). Nanocarriers as an emerging platform for cancer therapy. Nat Nanotechnol.

[14] Kim SW, Kim S, Tracy JB, Jasanoff A, Bawendi MG (2005). Phosphine oxide polymer for water-soluble nanoparticles. J Am Chem Soc.

[15] Clift MJ, Boyles MS, Brown DM, Stone V (2010). An investigation into the potential for different surface-coated quantum dots to cause oxidative stress and affect macrophage cell signalling in vitro. Nanotoxicology.

[16] Ho CC, Chang H, Tsai HT, Tsai MH, Yang CS, Ling YC (2013). Quantum dot 705, a cadmium-based nanoparticle, induces persistent inflammation and granuloma formation in the mouse lung. Nanotoxicology.

[17] Ho CC, Luo YH, Chuang TH, Yang CS, Ling YC, Lin P (2013). Quantum dots induced monocyte chemotactic protein-1 expression via MyD88-dependent toll-like receptor signaling pathways in macrophages. Toxicology.

[18] Zhang Y, Pan H, Zhang P, Gao N, Lin Y, Luo Z (2013). Functionalized quantum dots induce proinflammatory responses in vitro: the role of terminal functional group-associated endocytic pathways. Nanoscale.

[19] Liu L, Zhong X (2012). A general and reversible phase transfer strategy enabling nucleotides modified high-quality water-soluble nanocrystals. Chem Commun.

[20] Liu L, Guo X, Li Y, Zhong X (2010). Bifunctional multidentate ligand modified highly stable water-soluble quantum dots. Inorg Chem.

[21] Schmittgen TD, Livak KJ (2008). Analyzing real-time PCR data by the comparative CT method. Nat Protoc.

[22] Iwasaki A, Medzhitov R (2010). Regulation of adaptive immunity by the innate immune system. Science.

[23] Dwivedi PD, Tripathi A, Ansari KM, Shanker R, Das M (2011). Impact of nanoparticles on the immune system. J Biomed Nanotechnol.

[24] Nicolete R, dos Santos DF, Faccioli LH (2011). The uptake of PLGA micro or nanoparticles by macrophages provokes distinct in vitro inflammatory response. Int Immunopharmacol.

[25] Chen GY, Yang HJ, Lu CH, Chao YC, Hwang SM, Chen CL (2012). Simultaneous induction of autophagy and toll-like receptor signaling pathways by graphene oxide. Biomaterials.

[26] Romoser AA, Chen PL, Berg JM, Seabury C, Ivanov I, Criscitiello MF (2011). Quantum dots trigger immunomodulation of the NFκB pathway in human skin cells. Mol Immunol.

[27] Nagy A, Steinbrück A, Gao J, Doggett N, Hollingsworth JA, Iyer R (2012). Comprehensive analysis of the effects of CdSe quantum dot size, surface charge, and functionalization on primary human lung cells. ACS Nano.

[28] Zhang Y, Zhang Y, Hong G, He W, Zhou K, Yang K (2013). Biodistribution, pharmacokinetics and toxicology of Ag_2_S near-infrared quantum dots in mice. Biomaterials.

