# Supplementary material for: AMP-Conjugated Quantum Dots: Low Immunotoxicity Both In Vitro and In Vivo
Source: Nanoscale Res Lett. 2015 Nov 5;10:434. doi: 10.1186/s11671-015-1100-3 (PMC4635318; doi:10.1186/s11671-015-1100-3)
Supplement: Additional file 1: Figure S1. — Intracellular Cd2+ concentrations in macrophage J774A.1 cells incubated with quantum dots (50 nM) for 12 h were measured. Data are presented as means ± s.d. (n = 6). Figure S2. Colloidal stability of AMP-QDs in physiological media. AMP-QDs were incubated for 24 h in 0.01 M PBS (a) or cell culture medium containing 10 % foetal bovine serum, (b) and their size distributions were measured by a dynamic light scattering (DLS) with Zetasizer NanoZS Instrument. The hydrodynamic size of AMP-QDs in 0.01 M PBS (a) and cell culture medium (b) were determined to be 8.49 nm and 8.56 nm, respectively. [file 11671_2015_1100_MOESM1_ESM.docx]

Additional file 1

**AMP-conjugated quantum dots: low immunotoxicity both *in vitro* and *in vivo***

Tongcheng Dai^1^, Na Li^1^, Lu Liu,^3^ Qin Liu^1,2*^, Yuanxing Zhang^1,2^

^1^State Key Laboratory of Bioreactor Engineering, East China University of Science and Technology, Shanghai 200237, China

^2^Shanghai Collaborative Innovation Center for Biomanufacturing Technology, Shanghai 200237, China

^3^Department of Chemistry, College of Science, University of Shanghai for Science and Technology, Shanghai 200093, China





Figure S1 Intracellular Cd^2+^ concentrations in macrophage J774A.1 cells incubated with quantum dots (50 nM) for 12 h. Data are presented as means ± s.d. (n=6).


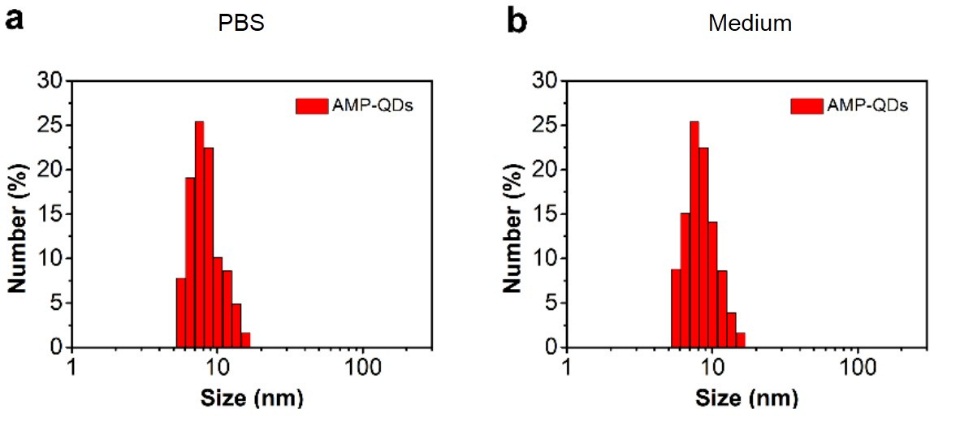


Figure S2 Colloidal stability of AMP-QDs in physiological media. AMP-QDs were incubated for 24 h in 0.01 M PBS (a) or cell culture medium containing 10% fetal bovine serum (b) and their size distribution were measured by a dynamic light scattering (DLS) with Zetasizer NanoZS Instrument. The hydradynamic size of AMP-QDs in 0.01 M PBS (a) and cell culture medium (b) was determined to be 8.49 nm and 8.56 nm respectively.
